# Topical and intravenous administration of human umbilical cord mesenchymal stem cells in patients with diabetic foot ulcer and peripheral arterial disease: a phase I pilot study with a 3-year follow-up

**DOI:** 10.1186/s13287-022-03143-0

**Published:** 2022-09-05

**Authors:** Che Zhang, Li Huang, Xiaofen Wang, Xiaoya Zhou, Xiaoxian Zhang, Ling Li, Jieying Wu, Meng Kou, Cheguo Cai, Qizhou Lian, Xihui Zhou

**Affiliations:** 1 Department of Pediatrics, The First Affiliated Hospital of Xi’an Jiaotong University, No. 277 West Yanta Road, Xi’an, 710061 Shaanxi China; 2 Clinical Research Centre, Affiliated Taihe Hospital of Hubei University of Medicine, Shiyan, China; 3 Guangzhou Cord Blood Bank, Guangzhou Women and Children’s Medical Center, Guangzhou Medical University, Guangzhou, 510623 China; 4 Department of Endocrinology, Affiliated Taihe Hospital of Hubei University of Medicine, Shiyan, China; 5 Clinical Data Center, Guangzhou Women and Children’s Medical Center, Guangzhou Medical University, Guangzhou, China; 6 Shenzhen Beike Biotechnology Co., Ltd., Shenzhen, China; 7 Department of Medicine, LKS Faculty of Medicine, The University of Hong Kong, Hong Kong SAR, China

**Keywords:** Diabetic foot ulcer, Diabetes complications, Human umbilical cord mesenchymal stem cells, Peripheral arterial disease

## Abstract

**Background:**

Diabetic foot ulcer (DFU) is a serious chronic complication of diabetes mellitus that contributes to 85% of nontraumatic lower extremity amputations in diabetic patients. Preliminary clinical benefits have been shown in treatments based on mesenchymal stem cells for patients with DFU or peripheral arterial disease (PAD). However, the long-term safety and benefits are unclear for patients with both DFU and PAD who are not amenable to surgical revascularization.

**Methods:**

In this phase I pilot study, 14 patients with PAD and incurable DFU were enrolled to assess the safety and efficacy of human umbilical cord mesenchymal stem cell (hUC-MSC) administration based on conservative treatments. All patients received topical and intravenous administrations of hUC-MSCs at a dosage of 2 × 10^5^ cells/kg with an upper limit of 1 × 10^7^ cells for each dose. The adverse events during treatment and follow-up were documented for safety assessments. The therapeutic efficacy was assessed by ulcer healing status, recurrence rate, and 3-year amputation-free rate in the follow-up phase.

**Results:**

The safety profiles were favorable. Only 2 cases of transient fever were observed within 3 days after transfusion and considered possibly related to hUC-MSC administration intravenously. Ulcer disclosure was achieved for more than 95% of the lesion area for all patients within 1.5 months after treatment. The symptoms of chronic limb ischaemia were alleviated along with a decrease in Wagner scores, Rutherford grades, and visual analogue scale scores. No direct evidence was observed to indicate the alleviation of the obstruction in the main vessels of target limbs based on computed tomography angiography. The duration of rehospitalization for DFU was 2.0 ± 0.6 years. All of the patients survived without amputation due to the recurrence of DFU within 3 years after treatments.

**Conclusions:**

Based on the current pilot study, the preliminary clinical benefits of hUC-MSCs on DFU healing were shown, including good tolerance, a shortened healing time to 1.5 months and a favorable 3-year amputation-free survival rate. The clinical evidence in the current study suggested a further phase I/II study with a larger patient population and a more rigorous design to explore the efficacy and mechanism of hUC-MSCs on DFU healing.

*Trial registration*: The current study was registered retrospectively on 22 Jan 2022 with the Chinese Clinical Trial Registry (ChiCTR2200055885), http://www.chictr.org.cn/showproj.aspx?proj=135888

**Graphical Abstract:**

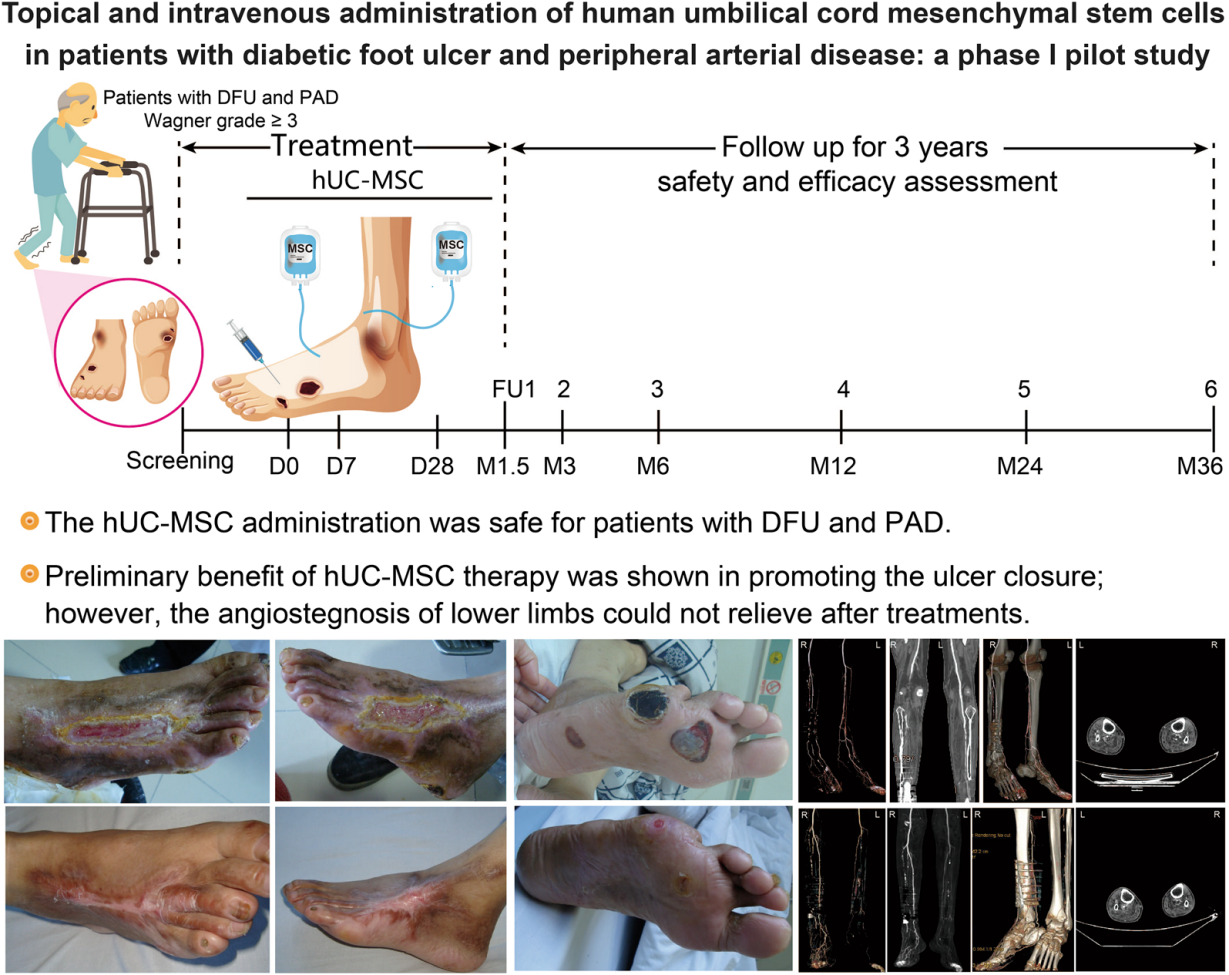

**Supplementary Information:**

The online version contains supplementary material available at 10.1186/s13287-022-03143-0.

## Background

Diabetic foot ulcer (DFU) is a serious chronic complication of diabetes mellitus (DM) with a considerable lifetime incidence (19%–34%) and high recurrence rate (40%–65%) in diabetic patients [1]. Typically, DFU is known as a precipitating factor in approximately 85% of cases of nontraumatic lower extremity amputations in diabetic patients [2]. The annual mortality in patients with incurable DFU is reported to be 11%, which rises to 22% among patients following incident lower-extremity amputation [3]. In particular, diabetic patients with concomitant peripheral arterial disease (PAD) [4] often require amputation for DFU and have a worse outcome, with a 5-year mortality of 50% [5]. Therefore, DFU has become a significant threat to public health and places a heavy burden on the economy and medical health system [6]. According to the 2019 report of the International Diabetes Federation, the direct costs of diabetes reach 760 billion USD globally [7], a substantial portion (30%–40%) of which is expended on medical care for DFU [8, 9]. Although surgical revascularization is recommended for patients with ulcers not healing after 4–6 weeks of routine treatments [5], a substantial portion of patients are not suitable for the surgery due to poor tolerance to cardiovascular or renal complications and arterial obstruction at the distal end of the lower extremity [10, 11].

In recent decades, novel therapeutic strategies based on mesenchymal stem cells (MSCs) have been designed to fill the unmet medical needs of patients with incurable DFUs. Emerging clinical trials focus mainly on adipose-derived mesenchymal stem cells (AD-MSCs; 54%, 14/26), bone marrow mesenchymal stem cells (BM-MSCs; 23%, 6/26), and umbilical cord mesenchymal stem cells (UC-MSCs; 12%, 3/26) in phase 1/2 (Clinicaltrials.gov). Accumulating clinical evidence has shown preliminary benefits of MSCs on wound closure, including the AD-MSC-hydrogel complex [12], BM-MSCs [13], and UC-MSCs [14]. Recently, UC-MSCs have attracted attention for clinical translation due to their better accessibility, higher proliferative potential, and lower immunogenicity than AD-MSCs and BM-MSCs. [15–17]. Previous studies indicated that patients with DM could benefit from intravenous transfusion of BM-MSCs [18] and UC-MSCs [19] with improvements in haemoglobin A1c (HbA1c). Preliminary clinical benefits and safety profiles were observed in a 3-month follow-up for patients with DFU who received both topical and intravenous transfusions of UC-MSCs after angioplasty [14]. In addition, the therapeutic potential of BM-MSCs was shown for patients with PAD after intramuscular injection in the ischaemic limb [20]. However, the safety, efficacy, and long-term benefits of UC-MSCs are unclear for patients with both serious DFU and PAD who are not amenable to surgical revascularization. In this phase I pilot study, we evaluated the safety and therapeutic benefits of UC-MSC administration in a combination of topical and intravenous routes based on a 3-year follow-up to provide new insights into the field of stem cell therapy for diabetic patients with incurable foot ulcers and PAD.

## Methods

### Study design and ethical approval

**Fig. 1:**
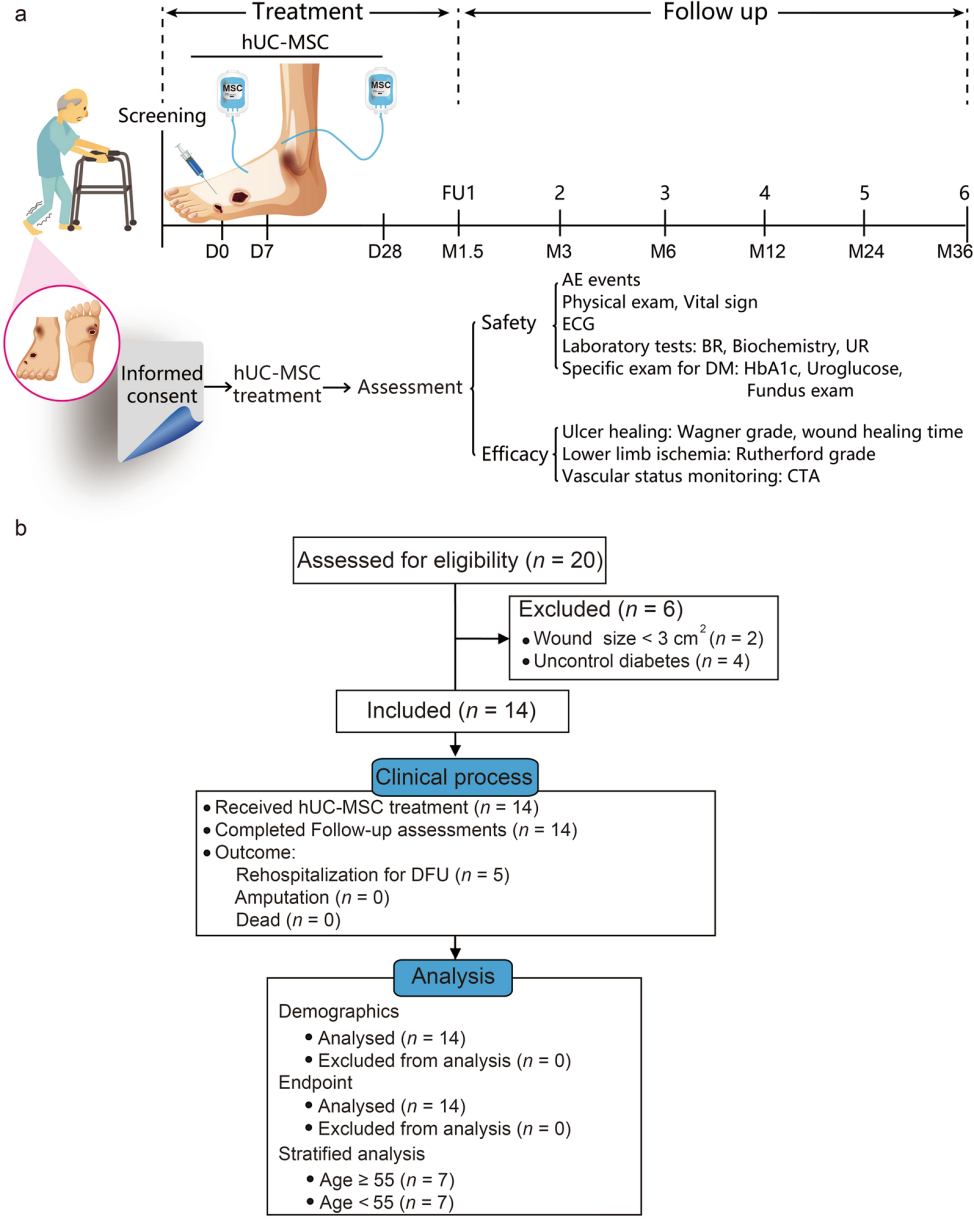
Flowchart of the study procedure. **a** The patients completed a long-term follow-up for 3 years after the last dose of hUC-MSCs and received laboratory tests, ulcer healing assessments, and vascular status monitoring for safety and efficacy evaluations. **b** Fourteen patients were included in the current study and completed the follow-up assessments

The current study was an open-label, single-arm, phase I pilot study to assess the safety and therapeutic potential of UC-MSCs in patients with incurable DFU and PAD. The eligible patients received 3 doses of hUC-MSC and basic treatments and then finished follow-up visits as scheduled until the study endpoint event was reached (death or lost to follow-up) or a 3-year follow-up visit was finished (Fig. 1a). The study was registered with Chictr.org.cn (ChiCTR2200055885). Ethical approval was obtained from the Institutional Review Board of the Affiliated Taihe Hospital of Hubei University of Medicine (ethical approval 20090903).

### Patient population

Patients were recruited from the Affiliated Taihe Hospital of Hubei University of Medicine and gave their written informed consent before any study-specific procedure. Patients who fulfilled the following criteria were included: (1) diagnosed with type 2 diabetes according to the World Health Organization (WHO) guidelines [21]; (2) diagnosed with PAD [22, 23]; (3) lower-limb ulcer not healing after 6 weeks of routine treatments; (4) wound size ≥ 3 cm^2^ (Additional file 1: Text S1); (5) Wagner grade [24, 25] ≥ 3 (Additional file  1: Table S1); (6) Rutherford category [26] ≥ 3 (Additional file 1: Table S2); (7) aged 18–80 years; (8) not amenable to surgical revascularization; and (9) written informed consent was obtained.

Patients with at least one of the below conditions were excluded: (1) uncontrol diabetes within 14 days before screening (HbA1c ≥ 8.0% for patients with DM history of 10 years or more; HbA1c ≥ 7.5% for patients with DM history less than 10 years) [27–29]; (2) serious allergy history or known allergy to more than 2 kinds of food or medications; (3) known allergy to stem cells or their derived products or vehicles; (4) serious renal complications (creatinine clearance rate < 30 ml/min); (5) serious liver dysfunction (ALT or AST > 3 times of upper limit of normal range confirmed on two consecutive measurements during the screening period); (6) CPK elevated > 3 times of upper limit of normal range at screening; (7) symptomatic congestive heart failure classified as class II–IV according to the New York Heart Association classification system at screening; (8) myocardial infarction, acute coronary syndrome or transient ischaemic stroke within 6 months prior to screening; (9) uncontrolled immunological disease or active serious systemic infection; (10) serious haematologic or coagulation disorder; (11) history of malignancy; (12) participation in other clinical trials within 3 months before screening; (13) treatments based on stem cells or their derived products received within 12 months before screening; and (14) any other concerns that hampered the compliance or safety as judged by the investigator.

The principal investigator confirmed the eligibility of candidates before enrolment. In particular, the risks and potential benefits for revascularization were evaluated by a multidisciplinary team (MDT) to determine whether the candidates were suitable for revascularization based on their comorbidities, complications, and status of wounds and ischaemia. This working group was formed by investigators from multidisciplinary departments, including endocrinologists, vascular surgeons, interventional radiologists, infectious disease physicians, orthopaedic surgeons, physiatrist, and pharmacists.

### Basic treatments

All patients received hypoglycaemic agents and treatments for their complications as well as symptomatic therapies, including debridement and antibiotics, in case of infection (Additional file 1: Table S3). The severity of infected ulcers was evaluated by the MDT with an infectious disease specialist based on the classification system of International Working Group on the Diabetic Foot (IWGDF) [30]. Antibiotics were selected empirically (Additional file 1: Table S4) according to the severity of infection until the results of drug sensitive tests were available. Sharp debridement was performed to remove the necrotic tissue and surrounding callus of the ulcers in accordance with the guidelines for the use of interventions to enhance the healing of DFUs [31]. Dressing was selected on the basis of exudate control, comfort, and cost [31]. Therefore, a basic wound contact dressing was used as a cost-effective alternative in our study to facilitate better exudate control and efficacy assessments (Additional file 1: Table S3). In particular, peripheral neuropathy was one of the most common comorbidities in patients with DFU. For patients with peripheral neuropathy, offloading devices were suggested in accordance with the recommendation in guidelines [32]. Removable ankle-high devices were more acceptable and chosen depending on the status of ulcers, including half shoes, forefoot offloading shoes, and custom-made shoes.

### Preparation and transplantation of hUC-MSCs

Allogeneic hUC-MSCs were obtained from Shenzhen Beike Biotechnology Company Co., Ltd. As described in our previous study [33], umbilical cord samples were donated from healthy puerperal women who were eligible for donation according to the requirements of the American Association of Blood Banks [34] after they provided informed consent. In brief, Wharton’s jelly was separated from pieces of umbilical cord samples and exposed for incubation after removing vessels. The isolated cells were cultivated in serum-free Dulbecco’s modified Eagle’s medium (DMEM) supplemented with cytokines and harvested at passage 4. The harvested cells showed the potential to differentiate into osteoblasts and adipocytes and were tested for high expression of MSC-specific surface markers (> 95%) and negative expression of haematopoietic stem cell-specific markers (< 2%) by a flow cytometer (FACSCalibur, BD Biosciences, USA). Quality tests were conducted on the cell products before clinical usage (Table 1) according to the International Society for Cellular Therapy Standards [35].

**Table 1:** Characteristics of human umbilical cord mesenchymal stem cells for treatments

Quality parameters	Results (*n* = 4 ^a^)
Cell surface markers	
HLA-DR, mean ± SD (%)	1.0 ± 0.2
CD79a/ CD19, mean ± SD (%)	0.8 ± 0.2
CD45, mean ± SD (%)	1.5 ± 0.3
CD34, mean ± SD (%)	1.3 ± 0.4
CD14, mean ± SD (%)	1.1 ± 0.3
CD105, mean ± SD (%)	98.4 ± 0.3
CD90, mean ± SD (%)	98.7 ± 0.6
CD73, mean ± SD (%)	97.3 ± 0.4
Viability rate, mean ± SD (%)	98.2 ± 0.3
Cell count, cells/ ml	(2.1 ± 0.1) × 10^7^
Pathogen tests	
Anaerobic bacteria	Negative
Aerobic bacteria	Negative
Fungi	Negative
Cytomegalovirus	Negative
Human T-cell leukemia virus	Negative
Mycoplasma	Negative
Endotoxin	< 0.5 EU/ ml

^a^hUC-MSCs were provided in 4 batches

*HLA-DR* human leukocyte antigen-DR, *hUC-MSC* human umbilical cord mesenchymal stem cell, *SD* standard deviation

All eligible patients received 3 doses of hUC-MSCs after enrolment without pretreatment with immune suppressants based on the safety profiles in our previous studies [33, 36]. Topical administration was an efficient way to deliver MSCs to the target tissue surrounding the ulcers, while preliminary benefits of intravenous administration of MSCs to limb ischaemia were shown in preclinical studies [37, 38] when the study was designed. Therefore, topical and intravenous administrations were performed successively for all patients, considering that both DFU and PAD existed in those patients. hUC-MSCs were injected subcutaneously into the tissues surrounding the periphery of the lesion, delivered into the base of ulcers on Day 0, and then infused intravenously into the target foot on Day 7 (Fig. 1a). Finally, the third dose of hUC-MSCs was infused intravenously at an interval of 28 days after the first dose to reduce the risk of cell embolism for repeated administrations in target limbs. Current recommendations for a single dose of UC-MSCs were 5 × 10^5^ cells/kg by intravenous infusion for neurorestoration, which could be reduced to 1/2 for elderly patients [39]. This was taken into consideration for dosage setting as peripheral neuropathy was common in patients with DFU. In addition, the number of cells in a safe range was indicated as 1–5 × 10^7^ cells during intramuscular injection or intra-arterial injection for DFU treatment. [40]. Therefore, the dosage was determined by the weight of the patient as 2 × 10^5^ cells/kg with an upper limit of 1 × 10^7^ cells. For patients whose weight were calculated as or more than 50 kg, the dosage was fixed to 1 × 10^7^ cells. For safety reasons, an individual dosage would be calculated based on a weight less than 50 kg in the case of emaciation due to DM. For the initial treatment, an hUC-MSC suspension with normal saline (20 ml) was administered in a series of injections to the edge and the base of ulcers with a small volume (0.5–1.0 ml in one injection per 1.5 cm^2^). Successive infusions of hUC-MSCs were given intravenously to the target foot for 15–20 min. The injection sites for intravenous administration were selected away from the ulcers and surrounding regions. Superficial veins in target feet were preferred for injection, including the great saphenous vein, dorsal venous arch and dorsalis pedis vein in preference. The administration of hUC-MSCs was conducted under close safety monitoring in a therapeutic room with rescue facilities in the Cell Therapy Center of the hospital.

### Data collection and clinical assessments

Clinical data were retrieved from medical charts, including demographics, medical history, and concomitant medication. The collected data were cross-checked by two study staff for quality control. Physical exams, fundus exams, and electrocardiograms (ECGs) as well as laboratory tests, including haematological tests, serum biochemistry, serologic assays, HbA1c, and routine urine tests, were performed (Table 2). In addition, the severity of ulcers was evaluated with the Wagner grade system [24, 25] (Additional file 1: Table S1), while the ischaemic status of the lower limb was assessed with the Rutherford category system to evaluate the severity of PAD [26] (Additional file 1: Table S2). The vascular status of the target foot was examined by computed tomography angiography (CTA). The relief of pain was assessed with a visual analogue scale [41, 42] (VAS, Additional file 1: Text S2).

**Table 2:** Study schedule and procedure

Study items	Screening	Treatment			Follow-up					
		Inj 1	Inj 2	Inj 3	FU1	FU2	FU3	FU4	FU5	FU6
		D 0	D 7	D 28	M 1.5	M 3	M 6	M12	M24	M36
Informed consent	×									
Medical history	×				×	×	×	×	×	×
Concomitant medicine	×	×	×	×	×	×	×	×	×	×
AE	×	×	×	×	×	×	×	×	×	×
PE	×	×	×	×	×	×	×	×	×	×
Vital signs	×	×	×	×	×	×	×	×	×	×
Fundus exam	×						×	×	×	×
Wagner grade	×		×	×	×	×	×	×		
Rutherford grade	×		×	×	×	×	×	×		
CTA	×				×			×		
ECG	×		×	×	×	×	×	×	×	×
BR	×		×	×	×	×	×	×	×	×
Biochemistry	×		×	×	×	×	×	×	×	×
HbA1c	×				×	×	×	×	×	×
Uroglucose	×		×	×	×	×	×	×	×	×
UR	×		×	×	×	×	×	×	×	×
Serologic assays ^a^	×									
hUC-MSC injection		×	×	×						

^a^Serologic assays were performed in screening visit, including: detection for anti-HAV, anti-HBc, anti-HBe, anti-HBs, HBsAg, HBeAg, anti-HCV, and anti-HIV; FTA-ABS test; procalcitonin, CRP, ESR, and anti-ENA antibody

*AE* adverse event, *anti-HBc* antibody to hepatitis B virus core antigen, *anti-HBe* antibody to hepatitis B virus e antigen; anti-HBs, hepatitis B virus surface antibody; BR, blood routine test; CRP, C-reactive protein; CTA, computed tomography angiography; ECG, electrocardiogram; ENA, extractable nuclear antigen; ESR, erythrocyte sedimentation rate; FTA-ABS, fluorescent treponemal antibody absorption; HbA1c, hemoglobin A1c; hUC-MSC, human umbilical cord mesenchymal stem cell; HAV, hepatitis A virus; HBeAg, hepatitis B virus e antigen; HBsAg, hepatitis B virus surface antigen; HCV, hepatitis C virus; HIV, human immunodeficiency virus; Inj, injection, PE, physical exams; UR, urine routine test

### Study endpoints

The primary end-point of this study was the safety of hUC-MSC therapies on DFU, which was evaluated by the incidence of adverse events (AEs) relative to hUC-MSC administration and laboratory tests in the short-term (1.5 months) and long-term (3 years) follow-up. The secondary endpoint was the therapeutic potential of hUC-MSC therapy, which was assessed by the closure rate and healing duration of ulcers, ischaemia and angiostenosis status of the target limbs, rehospitalization duration, amputation rate, and survival within 3 years posttreatment.

### Statistical analysis

Clinical data were handled according to the analysis plan (Fig. 1b). Categorical variables are presented as frequency rates and numbers. Continuous variables are presented as the mean ± standard error or the median (interquartile range). No imputation was used for missing data. Nonparametric tests were used for uncertain population distribution with small samples. A two-tailed, paired-sample T test was used to compare continuous variables between baseline and follow-up. Statistical significance was considered if *P* < 0.05. Statistical analysis was conducted with SPSS software (version 20.0, International Business Machines Corporation, Armonk, NY, USA).

## Results

### Patient characteristics

From Sep 2009 to Sep 2018, 20 patients with incurable DFUs were assessed for eligibility after informed consent was obtained. Six candidates were excluded due to failure to meet requirements, including wound size and glycaemic control (Fig. 1b). Finally, 14 patients were enrolled with a median age of 54.0 (47.2–65.7) years, including 11 males and 3 females. The final follow-up visit was completed by March 2, 2021. A long course of the disease was noted as 9.0 (4.7–12.2) years. Hypertension (43%) and DM-related comorbidities, including diabetic peripheral neuropathy (64%), diabetic nephropathy (50%), and diabetic retinopathy (21%), were common in these patients. In addition, knee disarticulation was performed on the right lower limb of one patient due to diabetic foot 5 years before screening (Table 3).

**Table 3:** Clinical characteristics and outcomes of patients

Clinical features	All (*n* = 14)	Age ≥ 55 (*n* = 7)	Age < 55 (*n* = 7)
Demographics			
Gender			
Male, *n* (%)	11 (79)	6 (86)	5 (71)
Female, *n* (%)	3 (21)	1 (14)	2 (29)
Age, y			
Median (IQR), y	54.0 (47.2–65.7)	65.0 (56.0–75.0)	49.0 (39.0–53.0)
Duration since diagnosis of DM, y			
Median (IQR), y	9.0 (4.7–12.2)	11.0 (5.0–13.0)	6.0 (4.0–12.0)
HbA1c at baseline			
Median (IQR), %	7.3 (6.9–7.9)	7.0 (6.5–7.8)	7.6 (7.0–8.3)
Main comorbidities			
Diabetic peripheral neuropathy, *n* (%)	9 (64)	5 (71)	4 (57)
Diabetic nephropathy, *n* (%)	7 (50)	4 (57)	3 (43)
Diabetic retinopathy, *n* (%)	3 (21)	2 (29)	1 (14)
Hypertension, *n* (%)	6 (43)	4 (57)	2 (29)
Cerebrovascular disease, *n* (%)	4 (29)	3 (43)	1 (14)
Amputation history, *n* (%)	1 (7)	1 (14)	0
Outcomes			
Ulcer status at 1.5-month FU ^a^			
Complete closure, *n* (%)	14 (93)	7 (88)	7 (100)
Incomplete closure ^b^, *n* (%)	1 (7)	1 (12)	0
Rehospitalization for DFU			
Proportion, *n* (%)	5 (36)	3 (43)	2 (29)
Mean ± SD, y	2.0 ± 0.6	1.8 ± 0.7	2.3 ± 0.2
Amputation, *n* (%)	1 (7)	1 (14)	0
First amputation interval, y	3.6	3.6	NA
Amputation plane	Midfoot	Midfoot	NA
Survival at 3-year FU, *n* (%)	14 (100)	7 (100)	7 (100)

^a^There were 15 ulcers for assessments since one patient had two ulcers for treatments

^b^The closure area could not achieved 100% of the lesion area

*DFU* diabetic foot ulcer, *DM* diabetes mellitus, *FU* follow-up, *IQR* interquartile range, *SD* standard deviation

### Treatments

The dosage of hUC-MSCs was fixed at 1 × 10^7^ cells as all patients were over 50 kg in weight. In addition to the basic treatments, the patients received 3 doses of hUC-MSCs (3 × 10^7^ cells in total), while one patient received 4 doses (4 × 10^7^ cells in total) due to two ulcers existing in the target limb. For this patient, 2 doses of hUC-MSCs were given for the topical treatment of ulcers on 2 consecutive days (Additional file 1: Table S5), followed by intravenous transfusion in the target limb with ischaemia on Day 7 and Day 28.

### Outcomes

**Fig. 2:**
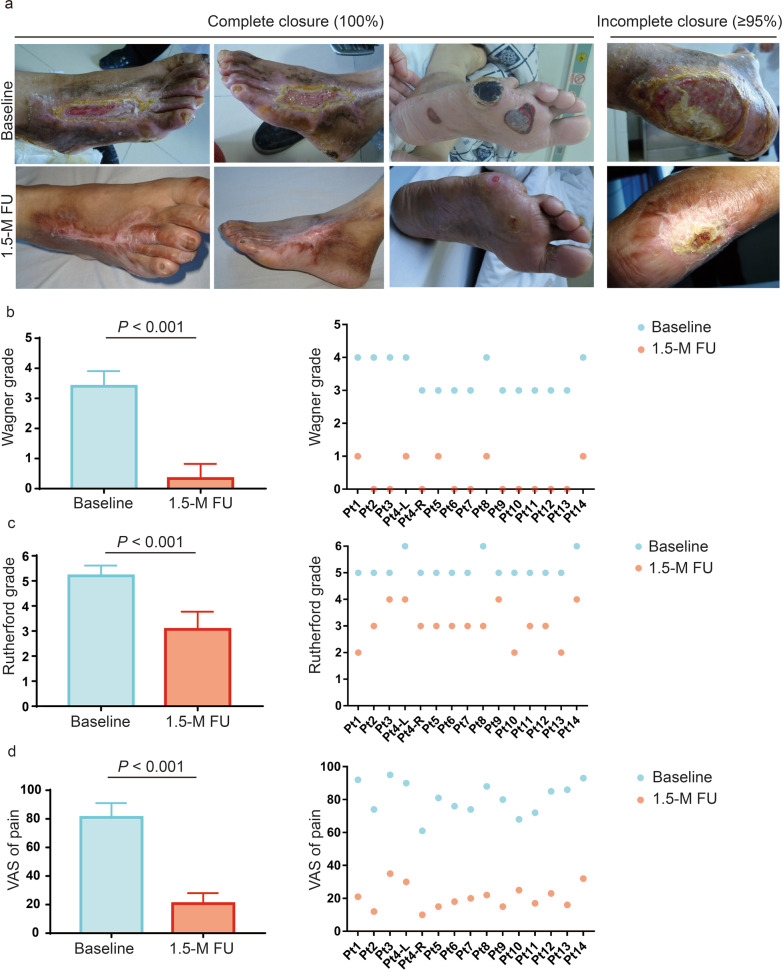
The remission of clinical symptoms in patients with diabetic foot ulcers at 1.5 months posttreatment. **a** The closure status of the diabetic foot ulcers was assessed at baseline and 1.5 months after treatments. Complete closure was observed for 14 ulcers out of 15, while incomplete closure was identified for one ulcer with a closure area of over 95%. **b** The severity grades of ulcers were decreased significantly after treatments based on the Wagner scores (*P* = 0.001). **c** The symptoms of chronic limb ischaemia were alleviated based on the Rutherford grades (*P* = 0.003). **d** The pain of the lower limbs was relieved remarkably at the 1.5-month follow-up based on the VAS assessments (*P* < 0.001)

**Fig. 3:**
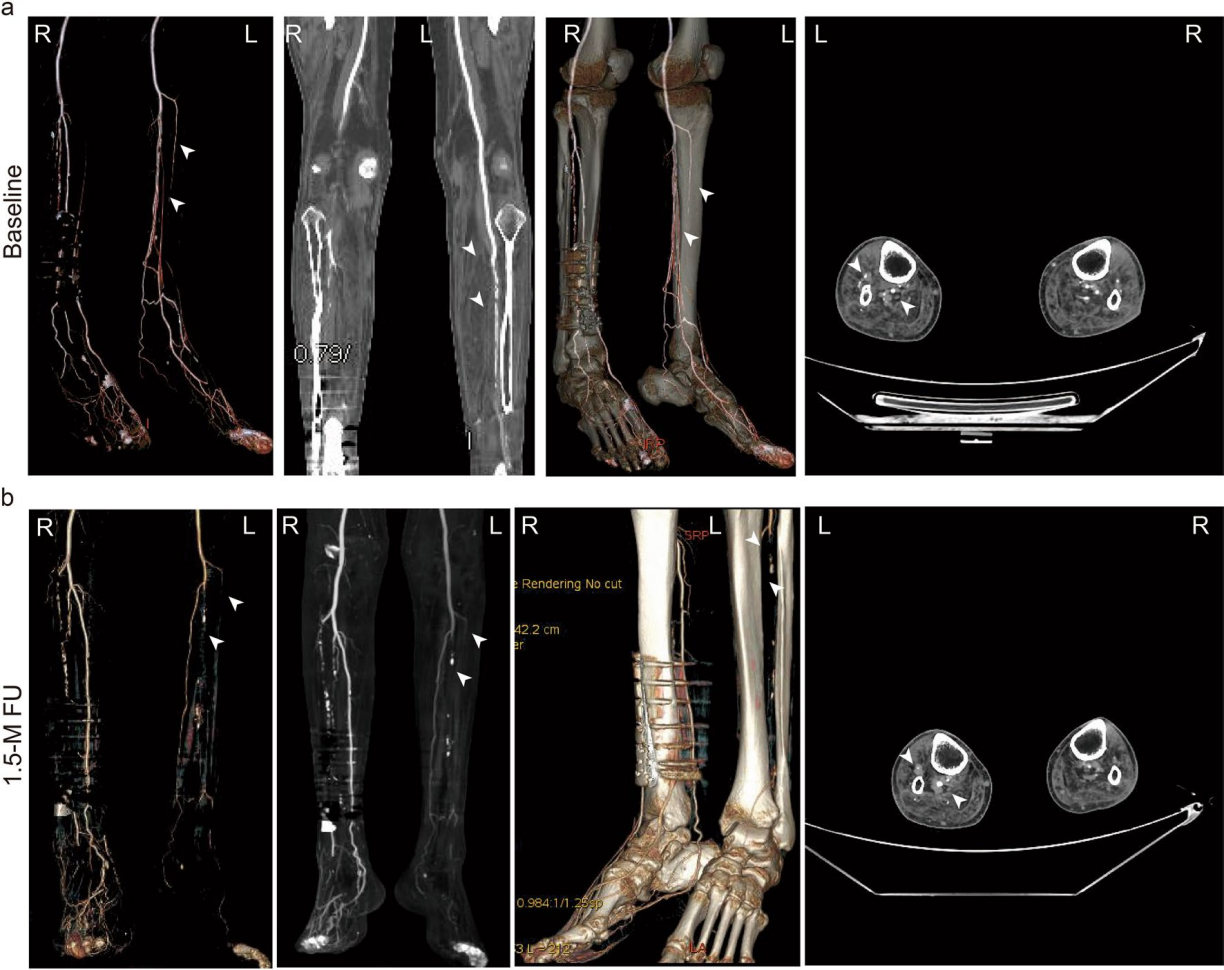
Typical CTA images of the lower limbs of the patients. The severity of angiostenosis of the target lower limbs could not be alleviated after treatments based on the CTA images of the patients. Typical images are shown below for Pt 01 at baseline (**a**) and at the 1.5-month follow-up (**b**). Angiostenosis was detected in anterior and posterior tibial arteries as well as arteriae fibularis for Pt 01, especially for anterior tibial artery and arteriae fibularis of his left lower limb (white arrows). No significant improvement was observed in the angiostenosis status of these arteries, in which calcification was detected (white arrows in figures of cross section), at 1.5 months after treatments

Based on the safety profile in a short-term follow-up at 1.5 months post-treatment, 2 cases of transient fever were considered possibly related to hUC-MSC transfusion intravenously, which were observed after transfusion and lasted for 1.5 ± 0.7 days with the highest temperatures of 38.4 °C and 38.5 °C, respectively. The patients recovered without treatment. In addition, diarrhoea, acute upper respiratory infection, oral ulcers, and arthrolithiasis were also reported, and they were mild or moderate and considered not related to the hUC-MSC treatments (Table 4). During the long-term follow-up, no hUC-MSC-related AEs or serious AEs were observed. No emerging complications were detected according to the laboratory tests, ECG, or fundus exams. In particular, the levels of HbA1c were stable in scheduled visits, indicating a stable status of diabetes. The closure status of ulcers was evaluated at the 1.5-month follow-up (Fig. 2a), and complete closure was observed in 14 out of 15 ulcers (80%). The closure area in one ulcer covered more than 95% of the lesion area, although it was considered an incomplete closure (Table 3). The severity grades of ulcers also decreased remarkably from baseline according to the Wagner scores (*P* = 0.001, Fig. 2b). Accordingly, the symptoms of chronic limb ischaemia were alleviated based on the Rutherford grades (*P* = 0.003, Fig. 2c) and VAS scores (*P* < 0.001, Fig. 2d), which indicated clinical symptomatic alleviation for PAD. However, no direct tomographic evidence supported a significant alleviation of the obstruction in the main vessels of the target limbs based on the CTA images of the patients at 1.5 months after treatment (Fig. 3). Despite limited benefits on the vascular recanalization of the legs, the long-term outcomes were favorable for ulcer disclosure in these patients. The duration of rehospitalization for DFU was 2.0 ± 0.6 years. All of the patients survived without amputation within 3 years after treatment, although a midfoot amputation was performed for one patient at 3.6 years posttreatment in the extended follow-up study (Table 3).

**Table 4:** Summary of adverse events during the short-term safety follow-up (1.5-M FU)

Adverse events	Cases	Cases onset				Duration, d	Severity grade			Relation with the hUC-MSC transplantation	Outcome
		Before transplantation	3d^a^	7d^a^	45d^a^		Mild	Moderate	Severe		
Fever	2	0	2	0	0	1.5 ± 0.7 ^b^	2	0	0	Possible	Recover
Diarrhea	2	0	1	1	0	3.5 ± 0.7 ^b^	2	0	0	Not related	Recover
Acute upper respiratory infection	1	1	0	0	0	5.0	0	1	0	Not related	Recover
Oral ulcers	1	0	0	0	1	10.0	1	0	0	Not related	Recover
Arthrolithiasis	1	1	0	0	0	8.0	0	1	0	Not related	Improved

^a^Adverse events were calculated based on the onset date within the timeframe after transplantation

^b^mean ± standard deviation

*hUC-MSC* human umbilical cord mesenchymal stem cell; 1.5-M FU, 1.5-month follow-up

## Discussion

DFU wounds often culminate in hospitalization and amputations due to deterioration into chronic ulcers with recurrent inflammation, infection, and ulceration. In China, 19% of patients with DFU had an amputation in 2015 [43], resulting in a high cost of treatments and health care [6, 7]. Surgical revascularization has provided a beneficial option to promote the wound healing of DFU by improving the blood circulation in the legs with angiostenosis [44, 45]; however, it is not feasible for patients with poor tolerance to the surgery. In addition, several factors are involved in the pathogenesis of diabetic wounds despite haemostasis, including inflammation, neurotrophy disorder, and interruption of epithelialization [46]. Therefore, comprehensive therapeutic strategies based on MSC therapy have been suggested for better clinical benefits in the long run as MSCs have shown potential for wound healing in diabetic rats through anti-inflammation [47, 48], neurotrophy [49], angiogenesis [50], and reepithelialization [51]. Topical administration of MSC was indicated to be safe with promising efficacy for patients with DFU in previous studies [12, 13]; however, the long-term safety and benefits remained unknown. In this phase I pilot study, the short-term and long-term safety of hUC-MSC administration was assessed up to a 3-year duration for DFU patients who were not amenable to surgical revascularization. In addition, the therapeutic benefits were also evaluated for wound healing and prognosis in the long term to provide valuable clinical evidence for reference in further studies.

Based on previous studies, poor glycaemic control with a higher HbA1c level was considered associated with a higher risk for the recurrence of DFU [52], while improvements in HbA1c could be achieved in patients with DM after intravenous transfusion of MSC [18, 19]. In addition, the obstructions of the main vessels of the target limbs were relieved after intravenous injection of UC-MSCs in a previous study [14]. Therefore, both topical and intravenous administrations were employed in our study to explore the safety of this scheme for the patients as well as the efficacy of hUC-MSCs on DFU healing and PAD of the target limbs. In previous studies, MSC transplantation was safe for clinical use by topical injection [12, 13] for DFU patients and intravenous injection for DM patients [18, 19, 53]. In these studies, no AEs were observed [19], no significant difference was detected in AE incidence between the MSC-treated group and the control group [12], or the AEs were considered not related to MSC administration [13]; however, upper respiratory infection was observed in mesenchymal precursor cells-treated patients [18]. In the current study, 2 cases of transient fever were considered possibly related to intravenous hUC-MSC administration, and they were mild and resolved without specific treatments. Although not reported in previous studies in patients with DFU or DM, transient fever was observed within 24 h after hUC-MSC transfusion in our prior study on patients with cerebral palsy [33]. Therefore, transient fever was considered one of the expected and controllable AEs relative to hUC-MSC transfusion based on our findings. The safety profiles were favorable as the other AEs within the 1.5-month follow-up were considered not related to hUC-MSC administration, and no hUC-MSC-related AEs were observed during the 3-year follow-up.

The topical administration of MSCs was believed to be critical to improving DFU healing, including decreasing the median time to complete closure and increasing the ulcer healing rate [12, 13]. Anti-inflammation and immunomodulation were considered to play a critical role in the underlying mechanism, which was supported by the changes in inflammatory cytokines, T-lymphocytes, and natural killer cells in patients before and after treatments [54, 55]. In addition, a more rapid replacement of granulation tissue by epithelial tissue was noted by pathological assessment after MSC treatment [56], which indicated that the patient could benefit from the potential of MSCs for reepithelialization. Better vasoreactivity was also observed based on the laser Doppler and transcutaneous oxygen pressure (TcpO_2_) assessments in the patients after MSC treatment [57]. In the current study, the duration of ulcer closure was within 1.5 months and reduced significantly compared with the patient themselves before hUC-MSC administration, as all of the patients had suffered from ulcers for more than 6 weeks (1.5 months) at baseline. In addition, all of the wounds achieved closure in more than 95% of the lesion area, and the complete closure rate was 80% 1.5 months after treatment. Accordingly, the symptoms were alleviated with the relief of ulcers. Based on the duration and closure status of DFU healing, the patients benefitted from the therapeutic scheme in our study.

The therapeutic efficacy of MSCs on limb ischaemia is still under debate and could be impacted by many factors, including but not limited to the individual variety of patients, therapeutic dosage, administration route, and injection site [58]. New collateral vessels were detected by CTA 1 month after hUC-MSC injection intramuscularly in a case series study [54], which indicated that angiostenosis was alleviated. Similarly, the symptoms of limb ischaemia were relieved after intramuscular injection of MSC, although no significant improvements in ABPI or revascularization were detected [59]. However, few improvements were observed in the ankle-brachial pressure index (ABPI) and ankle pressure after intramuscular injection of MSCs in a controlled study [20]. Preliminary clinical benefits were observed in patients with DM after intravenous transfusion of MSC, including improvements in ABPI and lower limb electromyogram data [60]. As an alternative to intramuscular injection, intravenous administration was conducted in the current study to explore the feasibility and potential of hUC-MSCs on PAD as well as the relationship of DFU healing with revascularization in the target limb. However, no direct tomographic evidence of collateral vessel angiogenesis or vascular revascularization was detected by CTA in the main vessels of target limes at 1.5 months after treatment, although the Rutherford grades and rest pain scores decreased remarkably along with ulcer healing. Based on the data in the current study, the efficacy of hUC-MSCs on DFU healing might not rely on the revascularization of profundal veins. Examinations of microcirculation were suggested to be used as a valuable complement to CTA in further studies.

The long-term outcomes were also important for chronic disease management in the life cycle. The recurrence of DFU [61] after conservative treatments is common, with a high proportion of 40% within 1 year and 65% within 3 years [1]. In our study, rehospitalization for DFU recurrence was observed in one patient (7%) within 1 year posttreatment, and in 36% of patients within 3 years posttreatment. In addition, the 3-year amputation-free survival rate in all patients was favorable as no amputation due to the recurrence of DFU occurred within 3 years after treatments; however, a midfoot amputation was performed for one patient at 3.6 years post-treatment in the extended safety follow-up. A prior major amputation history was noted for this patient, which indicated that a prior major amputation could be a high-risk factor for reamputation. Therefore, a previous history of amputation should be considered as a factor for subcategories in further studies. Among the other risk factors for the outcomes of DFU, uncontrolled HbA1c levels also contributed to a poor prognosis in the long run. In previous studies, the HbA1c level was decreased after treatment with MSC in patients with poor glycaemic control at baseline [18, 19]. In our study, the HbA1c levels were stable at scheduled visits without significant decreases from baseline. Unlike in previous studies, patients with poor glycaemic control were excluded from our study, and they might be more sensitive to the therapeutic potential of MSCs on glycaemic control after intravenous administration.

The interpretation of our findings is limited by the small sample size due to the phase I pilot study design. A parallel control group was not employed in the current stage of the study based on ethical consideration for the risk of amputation in this patient population, but preliminary clinical benefits were indicated when compared with the ulcer healing status in these patients before hUC-MSC treatments. Balancing the safety and potential benefits, the patient population was enrolled with relatively good conditions under strict criteria in the current stage of the study, and this was attributed to the low recruitment rate in the study; a bias could be introduced when referring the data to a patient population with more complex conditions. An enlarged patient population with more complex medical conditions and a control group could be considered in a further phase I/II study based on the good tolerance of patients to the treatment scheme in this pilot study. In addition, more direct evidence should be obtained in further studies to explore the underlying mechanism of hUC-MSCs on DFU healing. Assessments of tropic microcirculation in the target foot with ulcers could be a valuable supplement to CTA in further research. More rigorous designs are needed in further studies to optimize the patient population by subcategories, including a previous history of amputation.

## Conclusions

In conclusion, our findings support the safety of topical and intravenous administrations of hUC-MSCs for patients with DFU and PAD. The symptoms of chronic limb ischaemia were alleviated with wound healing within 1.5 months after treatment, and this mainly relied on topical injection. The long-term outcomes were favorable in terms of the recurrence rate and amputation-free survival rate within 3 years after treatment.

## Supplementary Information

**Additional file 1: Table S1.** Wagner grades: a classification system for diabetic foot ulcer. **Table S2.** Rutherford category: Clinical categories of chronic limb ischemia. **Table S3.** Basic treatments. **Table S4.** The empirical regimens of antibiotics for infected ulcers. Table S5 hUC-MSC treatments. **Text S1.** Measurement methods of ulcer area. **Text S2.** Visual analogue scale (VAS).

## Data Availability

All data generated or analyzed during this study are included in this published article and its supplementary information files. The data that support the findings of this study are available from the corresponding author upon reasonable request.

## Abbreviations

ABPI: Ankle-brachial pressure index
AD-MSC: Adipose-derived mesenchymal stem cell
AE: Adverse event
BM-MSC: Bone marrow mesenchymal stem cell
CTA: Computed tomography angiography
DFU: Diabetic foot ulcer
DM: Diabetes mellitus
DMEM: Dulbecco’s modified Eagle’s medium
ECG: Electrocardiogram
HbA1c: Hemoglobin A1c
hUC-MSC: Human umbilical cord mesenchymal stem cell
IRB: The institutional review board
MSC: Mesenchymal stem cells
PAD: Peripheral arterial disease
TcpO_2_: Transcutaneous oxygen pressure
UC-MSC: Umbilical cord mesenchymal stem cell
VAS: Visual analogue scale
WHO: The World Health Organization
