# Supplementary material for: Topical and intravenous administration of human umbilical cord mesenchymal stem cells in patients with diabetic foot ulcer and peripheral arterial disease: a phase I pilot study with a 3-year follow-up
Source: Stem Cell Res Ther. 2022 Sep 5;13:451. doi: 10.1186/s13287-022-03143-0 (PMC9446755; doi:10.1186/s13287-022-03143-0)
Supplement: Supplementary file 1 — Additional file 1: Table S1. Wagner grades: a classification system for diabetic foot ulcer. Table S2. Rutherford category: Clinical categories of chronic limb ischemia. Table S3. Basic treatments. Table S4. The empirical regimens of antibiotics for infected ulcers. Table S5 hUC-MSC treatments. Text S1. Measurement methods of ulcer area. Text S2. Visual analogue scale (VAS). [file 13287_2022_3143_MOESM1_ESM.docx]

# Topical and intravenous administration of human umbilical cord mesenchymal stem cells in patients with diabetic foot ulcer and peripheral arterial disease: a phase I pilot study with a 3-year follow-up

Che Zhang, Li Huang, Xiaofen Wang, Xiaoya Zhou, Xiaoxian Zhang, Ling Li, Jieying Wu, Meng Kou, Cheguo Cai, Qizhou Lian, Xihui Zhou

**Supplementary materials**

**Table S1** Wagner grades: a classification system for diabetic foot ulcer

**Table S2** Rutherford category: Clinical categories of chronic limb ischemia

**Table S3** Basic treatments

**Table S4** The empirical regimens of antibiotics for infected ulcers

**Table S5** hUC-MSC treatments

**Text S1** Measurement methods of ulcer area

**Text S2** Visual analogue scale (VAS)

**Table S1** Wagner grades: a classification system for diabetic foot ulcer ^[1, 2]^.

| **Grade** | **Clinical features** |
| --- | --- |
| 0 | Pre- or post-ulcerative lesion |
| 1 | Partial/full-thickness ulcer |
| 2 | Probing to tendon or capsule |
| 3 | Deep with osteitis |
| 4 | Partial foot gangrene |
| 5 | Whole foot gangrene |

**Table S2** Rutherford category: Clinical categories of chronic limb ischemia ^[3]^.

| **Category** | **Clinical description** | **Objective criteria** |
| --- | --- | --- |
| 0 | Asymptomatic: no hemodynamically significant occlusive disease | Normal treadmill or reactive hyperemia test |
| 1 | Mild claudication | Completes treadmill exerciser;  AP after exercise > 50 mmHg, but at least 20 mmHg lower than resting value |
| 2 | Moderate claudication | Between categories 1 and 3 |
| 3 | Severe claudication | Cannot complete standard treadmill exercise and AP after exercise <50 mmHg |
| 4 | Ischemic rest pain | Resting AP <40 mmHg, fiat or barely pulsatile ankle or metatarsal PVR;  TP <30 mmHg |
| 5 | Minor tissue loss: nonhealing ulcer,  focal gangrene with diffuse pedal  ischemia | Resting AP <60 mmHg, ankle or metatarsal PVR fiat or barely pulsatile; TP <40 mmHg |
| 6 | Major tissue loss: extending above TM level, functional foot no longer salvageable | Same as category 5 |

AP, Ankle pressure; PVR, pulse volume recording; TP, toe pressure; TM, transmetatarsal.

**Table S3** Basic treatments

| **Treatments** | **Commonly used drugs or materials** |
| --- | --- |
| Hypoglycemic agents ^a^ | Insulin (in preference)  Combination with: metformin, α-glucosidase inhibitor (acarbose), DPP-4 inhibitor (saxagliptin, sitagliptin, alogliptin), SGLT-2 inhibitor (empagliflozin, dapagliflozin, canagliflozin) |
| Anti-infection | Antibiotics based on drug sensitive test |
| Neurotrophic agents | Vitamin B1, cobamamide, mecobalamin, epalrestat, lipoic acid |
| Vasodilator agents ^b^ | Panax notoginseng saponin injection, alprostadil |
| Maintaining homeostasis | Rectification based on the laboratory test to maintain acid-base balance, electrolyte balance, and circulation capacity |
| Basic wound contact dressing | Petrolatum gauze, paraffin gauze, absorbable dressing |
| Others | Traditional Chinese medicine |

^a^ Insulin was used in preference, and other agents were combined based on the medical status of patients including insulin sensitivity, comorbidities, and complications.

^b^ Vasodilator agents were used to improve blood circulation.

Abbreviations: DPP-4, dipeptidyl peptidase-4; SGLT-2, sodium-dependent glucose transporters-2

**Table S4** The empirical regimens of antibiotics for infected ulcers ^[4]^

| **Infection severity** ^a^ | **Conditions for consideration** | **Empirical regimens** |
| --- | --- | --- |
| Mild | No complicating features | Semisynthetic penicillinase-resistant penicillin, cephalosperin (1^st^ generation) |
|  | β-lactam allergy or intolerance | Clindamycin, macrolide |
|  | Recent antibiotic exposure | Amoxicillin/clavulanate, ampicillin/sulbactam, trimethoprim/sulfamethoxazole |
|  | High risk for MRSA | Linezolid, trimethoprim/sulfamethoxazole, macrolide |
| Moderate or severe | No complicating features | Amoxicillin/clavulanate, ampicillin/sulbactam, cephalosperin (2^nd^ /3^rd^ generation) |
|  | Recent antibiotics | Ticarcillin/clavulanate,  piperacillin/tazobactam, cephalosperin (3^rd^ generation) |
|  | Macerated ulcer or warm climate | Ticarcillin/clavulanate,  piperacillin/tazobactam, semisynthetic penicillinase-resistant penicillin and ceftazidime |
|  | Ischaemic limb/necrosis/gas  forming | Amoxicillin/clavulanate, ampicillin/sulbactam, ticarcillin/clavulanate,  piperacillin/tazobactam |
|  | MRSA risk factors | Consider adding, or substituting with, glycopeptides;  linezolid; daptomycin |
|  | Risk factors for resistant GNR | Carbapenems; aminoglycoside and colistin |

^a^ The severity of infection was evaluated based on the IWGDF classification system.

Abbreviations: GNR, gram-negative rod; MRSA, methicillin-resistant *Staphylococcus aureus*

**Table S5** hUC-MSC treatments.

| Pt No. | 1^st^ dose ^a^, cells | 2^nd^ dose, cells | 3^rd^ dose, cells | Implanted cells totally |
| --- | --- | --- | --- | --- |
| 1 | 1×10^7^ | 1×10^7^ | 1×10^7^ | 3×10^7^ |
| 2 | 1×10^7^ | 1×10^7^ | 1×10^7^ | 3×10^7^ |
| 3 | 1×10^7^ | 1×10^7^ | 1×10^7^ | 3×10^7^ |
| 4 ^b^ | 2×10^7^ | 1×10^7^ | 1×10^7^ | 4×10^7^ |
| 5 | 1×10^7^ | 1×10^7^ | 1×10^7^ | 3×10^7^ |
| 6 | 1×10^7^ | 1×10^7^ | 1×10^7^ | 3×10^7^ |
| 7 | 1×10^7^ | 1×10^7^ | 1×10^7^ | 3×10^7^ |
| 8 | 1×10^7^ | 1×10^7^ | 1×10^7^ | 3×10^7^ |
| 9 | 1×10^7^ | 1×10^7^ | 1×10^7^ | 3×10^7^ |
| 10 | 1×10^7^ | 1×10^7^ | 1×10^7^ | 3×10^7^ |
| 11 | 1×10^7^ | 1×10^7^ | 1×10^7^ | 3×10^7^ |
| 12 | 1×10^7^ | 1×10^7^ | 1×10^7^ | 3×10^7^ |
| 13 | 1×10^7^ | 1×10^7^ | 1×10^7^ | 3×10^7^ |
| 14 | 1×10^7^ | 1×10^7^ | 1×10^7^ | 3×10^7^ |

^a^ The 1^st^ dose of hUC-MSC was given topically, while the 2^nd^ and 3^rd^ doses were given intravenously.

^b^ For patient No.4, two doses of hUC-MSC were given topically to the two ulcers respectively in two successive days. So, there were 15 ulcers received treatments and follow-up assessments.

Abbreviations: hUC-MSC, human umbilical cord mesenchymal stem cell; Pt, patient.

**Text S1** Measurement methods of ulcer area

Ulcer area = length of a longest edge (a) × length of a longest edge at vertical (b)


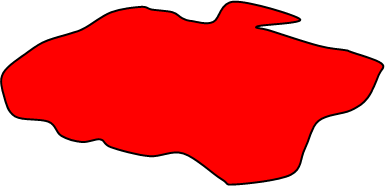


b

a

**Text S2** Visual analogue scale (VAS).

No pain Worst imaginable pain

|  |
| --- |
|  |

0 100mm

The patient is asked to mark a 100 mm line to indicate pain intensity. The score is measured from the zero anchor to the patient’s mark. Using a millimeter scale to measure the patient’s score will provide 101 levels of pain intensity ^[5, 6]^.

**References**

1. Wagner FJ. The dysvascular foot: a system for diagnosis and treatment. Foot Ankle. 1981;2(2):64-122.

2. Oyibo S, Jude E, Tarawneh I, Nguyen H, Harkless L, Boulton A. A comparison of two diabetic foot ulcer classification systems the Wagner and the University of Texas wound classification systems. Diabetes Care. 2001;24(1):84-8.

3. Rutherford R, Baker J, Ernst C, Johnston K, Porter J, Ahn S, et al. Recommended standards for reports dealing with lower extremity ischemia revised version. J Vasc Surg. 1997;26(3):517-38.

4. Lipsky BA, Senneville E, Abbas ZG, Aragon-Sanchez J, Diggle M, Embil JM, et al. Guidelines on the diagnosis and treatment of foot infection in persons with diabetes (IWGDF 2019 update). Diabetes Metab Res Rev. 2020;36 Suppl 1:e3280.

5. Williamson A, Hoggart B. Pain: a review of three commonly used pain rating scales. J Clin Nurs. 2005;14(7):798-804.

6. Peeters Weem SM, Teraa M, den Ruijter HM, de Borst GJ, Verhaar MC, Moll FL. Quality of life after treatment with autologous bone marrow derived cells in no option severe limb ischemia. Eur J Vasc Endovasc Surg. 2016;51(1):83-9.
